# How do ethnicity and deprivation impact on life expectancy at birth in people with serious mental illness? Observational study in the UK

**DOI:** 10.1017/S0033291720001087

**Published:** 2021-11

**Authors:** Jayati Das-Munshi, Chin-Kuo Chang, Alex Dregan, Stephani L. Hatch, Craig Morgan, Graham Thornicroft, Robert Stewart, Matthew Hotopf

**Affiliations:** 1 Institute of Psychiatry, Psychology & Neuroscience, King's College London, London, UK; 2 South London & Maudsley NHS Trust, London, UK; 3 ESRC Centre for Society and Mental Health, King’s College London, UK; 4 University of Taipei, Taipei, Taiwan

**Keywords:** Bipolar disorders, depression, deprivation, ethnicity, life expectancy, mortality, schizophrenia, severe mental illness

## Abstract

**Background:**

Across international contexts, people with serious mental illnesses (SMI) experience marked reductions in life expectancy at birth. The intersection of ethnicity and social deprivation on life expectancy in SMI is unclear. The aim of this study was to assess the impact of ethnicity and area-level deprivation on life expectancy at birth in SMI, defined as schizophrenia-spectrum disorders, bipolar disorders and depression, using data from London, UK.

**Methods:**

Abridged life tables to calculate life expectancy at birth, in a cohort with clinician-ascribed ICD-10 schizophrenia-spectrum disorders, bipolar disorders or depression, managed in secondary mental healthcare. Life expectancy in the study population with SMI was compared with life expectancy in the general population and with those residing in the most deprived areas in England.

**Results:**

Irrespective of ethnicity, people with SMI experienced marked reductions in life expectancy at birth compared with the general population; from 14.5 years loss in men with schizophrenia-spectrum and bipolar disorders, to 13.2 years in women. Similar reductions were noted for people with depression. Across all diagnoses, life expectancy at birth in people with SMI was lower than the general population residing in the most deprived areas in England.

**Conclusions:**

Irrespective of ethnicity, reductions in life expectancy at birth among people with SMI are worse than the general population residing in the most deprived areas in England. This trend in people with SMI is similar to groups who experience extreme social exclusion and marginalisation. Evidence-based interventions to tackle this mortality gap need to take this into account.

## Introduction

People with conditions such as schizophrenia or bipolar disorders experience marked reductions in life expectancy compared with the general population, ranging from 13 to 15 years (Hjorthøj, Stürup, McGrath, & Nordentoft, 2017) and in some contexts up to 20 years (Fekadu et al., 2018; Hjorthøj et al., 2017; Liu et al., 2017). This has been noted over time and is increasing (Lawrence, Hancock, & Kisely, 2013; Saha, Chant, & McGrath, 2007). There is some indication of marked heterogeneity in life expectancy in these populations internationally, with the largest reductions in life expectancy reported in studies from sub-Saharan Africa (Hjorthøj et al., 2017). A similar reduction has been reported for severe unipolar depression, ranging from 10 to 14-year reduction in life expectancy, compared with the general population (Laursen, Musliner, Benros, Vestergaard, & Munk-Olsen, 2016).

It remains unclear whether life expectancy in serious mental disorders varies by ethnic group and deprivation. In the general population, there is concern that inequalities in population-level life expectancy are widening over time, with the largest reductions in life expectancy noted in the most deprived communities across the UK, relative to the least deprived (Bennett et al., 2018). The largest contributors to shortened life expectancy in the general population are from the same common preventable physical health conditions, such as respiratory disorders and ischaemic heart disease, that account for the majority of deaths in people with serious mental illness (SMI) (Bennett et al., 2018; Das-Munshi et al., 2017b). Although the UK operates a system of universal access to healthcare it has been suggested that in deprived areas, access to healthcare may still be inequitable and may contribute to observed differences in life expectancy (Bennett et al., 2018). The interplay of these inequalities has not been previously assessed, despite a concern that access to care is also inequitable for people with SMI (Mitchell, Malone, & Doebbeling, 2018), fuelled by the stigma accorded to mental disorders (Thornicroft, 2018).

Common preventable conditions such as cardiovascular disease, type 2 diabetes mellitus and respiratory disorders account for the majority of deaths in people with serious mental disorders (Das-Munshi et al., 2017b; Saha et al., 2007). These are known to be elevated within certain ethnic minority groups, independent of the presence of serious mental illness (Health and Social Care Information Centre: HSIC, 2005). These conditions may become further elevated with the onset of mental illness (Das-Munshi et al., 2017a). Despite these important associations, there have been no studies which have examined differences in life expectancy at birth in SMI among ethnic minority groups (Hjorthøj et al., 2017).

We, therefore, aimed to assess variations in life expectancy at birth in a large ethnically diverse sample of people with schizophrenia-spectrum, bipolar disorders or depression, in contact with a secondary mental healthcare provider in London, UK. We included a sample of people with depression, as we considered that people in contact with secondary mental healthcare with depression represent the more ‘severe’ end of the spectrum since most ‘milder’ common mental disorders (including depression) are generally managed within primary care (Das-Munshi, Chang, Schofield, Stewart, & Prince, 2018). The sample of depressed individuals within this study may, therefore, represent complex cases referred to secondary mental healthcare for further management (Das-Munshi et al., 2018). We also sought to assess the moderating role of area-level deprivation and ethnicity on life expectancy at birth among men and women with SMI. ‘Serious mental illness’ was defined as including schizophrenia-spectrum, bipolar disorders as well as (severe) depression in contact with secondary care.

## Method

### Setting, participants and linkage to death certificates

We used electronic health records data from a large secondary care mental health service provider, South London & Maudsley NHS Foundation Trust (SLaM), providing near-complete secondary mental healthcare coverage to a well-defined catchment area covering approximately 1.3 million people in south-east London (Perera et al., 2016). Since 2006 SLaM has operated fully electronic health records for all its services. The SLaM Clinical Record Interactive Search (CRIS) system was established in 2007, allowing the search and retrieval of anonymised health records for the purposes of research (Perera et al., 2016).

Mental health clinicians are required to assign *International Classification of Mental Disorders-10* (*ICD-10*) (*World Health Organization*, 2011) codes for confirmed clinical diagnoses of mental healthcare service users. We used structured fields in CRIS, supplemented by natural language processing (NLP) algorithms developed through the Generalised Architecture for Text Engineering (GATE) software (Perera et al., 2016) to mine the free text in clinical records for diagnostic statements, supplementing clinician entries captured in structured fields (Perera et al., 2016). In previous work, these have been shown to enhance the detection of specific diagnoses of mental disorder with good sensitivity and positive predictive value for NLP-based approaches (Das-Munshi et al., 2018; Das-Munshi et al., 2019).

We thus identified ‘at risk’ cohorts of individuals with ICD-10 diagnoses spanning schizophrenia-spectrum (non-affective) disorders (ICD-10 codes: F2*), bipolar disorders (F30 and F31) and depression (F32 and F33). Individuals with a comorbid diagnosis of dementia (F0*) prior to the diagnosis of SMI or depression diagnoses were excluded. We developed a hierarchical approach whereby people with schizophrenia-spectrum or bipolar disorder diagnoses were given this diagnosis, irrespective of prior or later depression diagnosis and people in the depression group could not have any mention of bipolar disorders or schizophrenia-spectrum disorders. The samples used for this study have been previously described elsewhere (Das-Munshi et al., 2016; Das-Munshi et al., 2017b; Das-Munshi et al., 2018).

For inclusion into the study, individuals had to have a relevant clinical diagnosis before or between 1 January 2007 and 31 December 2014. Individuals were followed from diagnosis date until the earliest of either: death or the end of the study, on 31 December 2014. For individuals with a diagnosis before the window, entry date was set as 1 January 2007. For individuals with a diagnosis after this time, the entry date was set as the first recorded date of their diagnosis. We also extracted data on month and year of birth (to derive age for the sample), gender and ethnicity. Ethnicity was classified according to the Office for National Statistics (ONS) as White British, Black Caribbean, Black African and Irish. A ‘South Asian’ group which comprised the ethnic minority groups of Indian, Pakistani and Bangladeshi groups was deprived, due to very low numbers in each of these groups, not permitting separate analyses. Certain other groups were excluded (e.g. people of Chinese ethnicity) due to small numbers across samples and concerns related to disclosure risks. People identifying as of mixed ethnicity were grouped with the ethnic minority identity stated. Information on area of residence was derived by linking postcodes closest to the time of diagnosis to the Index of Multiple deprivation, a multi-domain small area assessment of deprivation (Noble, Wright, Smith, & Dibben, 2006) at lower super output area level. Lower super output areas are geographical areas of adjacent postcodes in the UK, which typically comprise a mean population of 1500 individuals.

### Death/mortality outcome

The primary outcome of the study was death from all causes. We used a linkage to death certificate information through the Office of National Statistics (ONS), to determine deaths and date of death, which was provided as a file for any deaths occurring over the observation period, in England and Wales.

### Statistical methods

Deaths by relevant ICD-10 diagnoses in men and women within the sample in 5 year age bands were used as an outcome measure for the analyses. As schizophrenia-spectrum and bipolar diagnoses are uncommon below the age of 15 years, we substituted under-15 years' mortality rates from the population of England and Wales from 2011, as this year was the midpoint of the cohorts; This methodology has been used previously for these populations (Chang et al., 2011). For comparability, we retained this approach for the sample with depression. We used Chiang's method of abridged life tables (Chiang, 1984), with 5 year age band up to age 85+ years, to estimate life expectancy at birth and associated standard errors/95% confidence intervals, using an Excel spreadsheet, provided and recommended by Public Health England. This approach is recommended for deriving life expectancy at birth for smaller study populations (Eayres & Williams, 2004). As we used a cohort with 8 years' data on follow-up and deaths, weights were then calculated taking the mean observation period contributed by individuals by age and sex, to derive the mean at-risk period for each age- and gender-band, applying to the denominator of each corresponding band.

Deaths and the ‘at risk’ population were used to estimate life expectancy at birth with 95% confidence intervals for men and women by ICD-10 diagnosis, and then according to ethnicity. Death summary tables were available from the UK Office for National Statistics (ONS) website (http://www.ons.gov.uk) and were used to compare life expectancy at birth in our study populations with mental illness against life expectancy in the general population from England and Wales and against life expectancy in the general population residing in the most deprived areas in England (defined as a resident in the most deprived decile according to the Index of Multiple Deprivation). These data were taken for the period 2011–2013 which was closest to the midpoint of our study cohort.

## Results

A total of 18 641 individuals contributed data to analyses relating to schizophrenia, schizoaffective, other schizophrenia-spectrum disorders and bipolar disorders and 20 203 individuals contributed data to analyses relating to depression. Table 1 highlights the demographic characteristics of the samples.

**Table 1.** Demographic overview

	Number	%
*Serious mental illness sample*^a^	18 641	
Age (years)^b^	44.5 (mean)	17.0 (s.d.)
Gender		
Male	9856	52.9%
Female	8785	47.1%
Ethnicity		
White British	9278	49.8%
Irish	565	3.0%
Black Caribbean	4946	26.5%
Black African	2573	13.8%
South Asian	1279	6.9%
Area-level deprivation (increasing deprivation, cut at decile)		
1st (Most deprived)	1824	9.8%
2^nd^	1925	10.3%
3^rd^	1831	9.8%
4^th^	1881	10.1%
5^th^	1829	9.8%
6^th^	1896	10.2%
7^th^	1878	10.1%
8^th^	1893	10.2%
9^th^	1879	10.1%
10^th^ (Least deprived)	1805	9.7%
Diagnosis		
Non-affective (Schizophrenia-spectrum)	13 476	72.3%
Affective (Bipolar disorders)	5165	27.7%
*Depression sample*^c^	20 203	
Age (years)^b^	46.3 (mean)	19.5 (s.d.)
Gender		
Male	7634	37.8%
Female	12 569	62.2%
Ethnicity		
White British	13 903	68.8%
Irish	754	3.7%
Black Caribbean	2942	14.6%
Black African	1863	9.2%
South Asian	741	3.7%
Area-level deprivation (increasing deprivation cut at decile)		
1^st^ (Most deprived)	2000	9.9%
2^nd^	2040	10.10%
3^rd^	2012	9.96%
4^th^	2004	9.92%
5^th^	2040	10.10%
6^th^	2025	10.02%
7^th^	2005	9.92%
8^th^	2010	9.95%
9^th^	2033	10.06%
10^th^ (Least deprived)	2034	10.07%

a Serious mental illness includes schizophrenia-spectrum (F2*) and bipolar disorders (F30,F31).

b Age on 1 January 2011 (study start date).

c Depression sample includes depression (F32, F33).

Relative to the general population in England, life expectancy at birth among people with any SMI diagnoses was markedly lower. Among men, life expectancy at birth was on average 12.6 years (for bipolar disorders) to 15.0 years (schizophrenia-spectrum and depressive disorders) lower compared to the life expectancy at birth among the general population; while among women, this was just over 13 years lower compared to women in the general population, across all serious mental disorder diagnoses assessed (schizophrenia-spectrum disorders, bipolar disorders and depression; see Tables 2 and 3). Across men and women with all SMI diagnoses, life expectancy at birth remained considerably lower than that of the general population residing in the most deprived areas nationally (Tables 2 and 3). All ethnic groups (including the White British group), across all mental illness diagnoses, experienced reductions in life expectancy at birth compared to the general population. The largest reductions in life expectancy were observed within schizophrenia-spectrum disorders for Irish men with 20.5 years lost, and in White British women with 16.3 years lost, compared to the general population. However, across some subgroups, 95% confidence intervals overlapped, indicating that smaller samples may have impacted precision.

**Table 2.** Life expectancy at birth for serious mental disorders by diagnosis and ethnicity: men

Diagnosis	Ethnicity	Observed deaths/total	Life expectancy (95% CI)	Difference from the male general population^a^	Difference from male general population living in the poorest areas^b^
Any serious mental illness	Total sample	909/9695	64.7 (63.0–66.4)	−14.5	−9.4
	White British	583/4724	62.3 (60.3–64.3)	−16.9	−11.8
	Black Caribbean	167/2651	70.6 (67.3–73.8)	−8.6	−3.5
	Black African	51/1337	68.6 (63.9–73.3)^c^	−10.6	−5.5
	South Asian	55/681	65.8 (61.5–70.1)	−13.4	−8.3
	Irish	52/302	61.8 (55.0–68.5)	−17.4	−12.3
Non-affective (schizophrenia-spectrum)	Total sample	732/7645	64.2 (62.4–66.0)	−15.0	−9.9
	White British	442/3341	61.3 (59.0–63.7)	−17.9	−12.8
	Black Caribbean	152/2363	70.1 (66.8–73.3)	−9.1	−4.0
	Black African	46/1196	70.6 (65.9–75.3)^c^	−8.6	−3.5
	South Asian	47/528	64.8 (60.0–69.6)	−14.4	−9.3
	Irish	45/217	58.7 (50.3–67.2)	−20.5	−15.4
Affective (bipolar disorders)	Total sample	177/2050	66.6 (64.0–69.3)	−12.6	−7.5
	White British	141/1383	65.0 (61.9–68.1)	−14.2	−9.1
	Black Caribbean	15/288	72.1 (66.0–78.1)	−7.1	−2.0
	Black African	5/141	68.6 (60.8–76.3)	−10.6	−5.5
	South Asian	9/153	72.6 (63.8–81.5)	−6.6	−1.5
	Irish	7/85	69.2 (61.9–76.4)	−10.0	−4.9
Depression	Total sample	1040/7634	64.2 (62.4–65.9)	−15.0	−9.9
	White British	857/5634	63.6 (61.8–65.5)	−15.6	−10.5
	Black Caribbean	74/806	66.8 (62.7–71.0)	−12.4	−7.3
	Black African	22/598	66.3 (60.7–71.9)	−12.9	−7.8
	South Asian	27/260	63.9 (57.1–70.7)	−15.3	−10.2
	Irish	60/336	64.7 (60.0–69.5)	−14.5	−9.4

a Life expectancy at birth in England for males (based on data for years 2011–2013): 79.2 years.

b Defined as residing in the most deprived decile of the Index of Multiple Deprivation, 2011–2013, England: 74.1 years.

c 0 deaths in final band therefore estimates from full sample inserted here.

**Table 3.** Life expectancy at birth for serious mental disorders by diagnosis and ethnicity: Women

Diagnosis	Ethnicity	Observed deaths/total	Life expectancy (95% CI)	Difference from the female general population^a^	Difference from female general population living in the poorest areas^b^
Any serious mental illness	Total sample	859/8640	69.8 (68.0–71.6)	−13.2	−9.3
	White British	547/4376	68.0 (65.9–70.1)	−15.0	−11.1
	Black Caribbean	165/2224	73.8 (69.9–77.7)	−9.2	−5.3
	Black African	56/1200	70.5 (67.0–74.0)^c^	−12.5	−8.6
	South Asian	39/583	75.6 (71.7–79.4)	−7.4	−3.5
	Irish	52/257	71.2 (66.8–75.6)	−11.8	−7.9
Non-affective (schizophrenia-spectrum)	Total sample	627/5621	69.7 (67.8–71.7)	−13.3	−9.4
	White British	377/2332	66.7 (64.1–69.3)	−16.3	−12.4
	Black Caribbean	134/1732	75.1 (70.6–79.6)	−7.9	−4.0
	Black African	49/987	70.6 (66.8–74.4)	−12.4	−8.5
	South Asian	31/432	75.1 (70.6–79.7)	−7.9	−4.0
	Irish	36/138	68.2 (61.1–75.3)	−14.8	−10.9
Affective (bipolar disorders)	Total sample	232/3019	69.9 (67.5–72.2)	−13.1	−9.2
	White British	170/2044	69.5 (66.8–72.3)	−13.5	−9.6
	Black Caribbean	31/492	67.7 (61.6–73.8)	−15.3	−11.4
	Black African	7/213	73.6 (67.8–79.4)	−9.4	−5.5
	South Asian	8/151	75.2 (68.7–81.7)	−7.8	−3.9
	Irish	16/119	72.1 (66.0–78.2)	−10.9	−7.0
Depression	Total sample	1321/12 569	69.9 (68.2–71.6)	−13.1	−9.2
	White British	1109/ 8269	68.6 (66.7–70.4)	−14.4	−11.3
	Black Caribbean	79/2136	75.3 (71.5–79.1)	−7.7	−3.8
	Black African	23/1265	77.8 (68.7–87.0)	−5.2	−1.3
	South Asian	26/481	72.3 (65.6–79.1)	−10.7	−6.8
	Irish	84/418	68.4 (64.4–72.5)	−14.6	−10.7

a Life expectancy at birth in England for females (based on data for years 2011–2013): 83.0 years.

b Defined as residing in the most deprived decile of the Index of Multiple Deprivation, 2011–2013, England: 79.1 years.

c 0 deaths in final band therefore estimates from full sample inserted here.

In general, trends were indicative of reduced life expectancy at birth among people with SMIs, irrespective of their ethnicity (Tables 2 and 3). In men with SMIs (defined as schizophrenia-spectrum or bipolar disorders combined) differences compared to the general population ranged from an 8.6 year (Black Caribbean) to a 17.4 year (Irish) reduction in life expectancy and in women this ranged from a 7.4 year (South Asian) to a 15.0 year (White British) reduction in life expectancy. A similarly adverse picture with respect to premature mortality was evident, with men with depression in contact with mental healthcare services experiencing reductions in life expectancy at birth ranging from 12.4 years (Black African) to 15.6 years (White British) and women with depression in contact with mental healthcare services experiencing reductions in life expectancy at birth ranging from 7.7 years (Black African) to 14.6 years (Irish), thus indicating a similar impact on life expectancy at birth across diagnostic groups as well as by ethnicity.

**Fig. 1.**
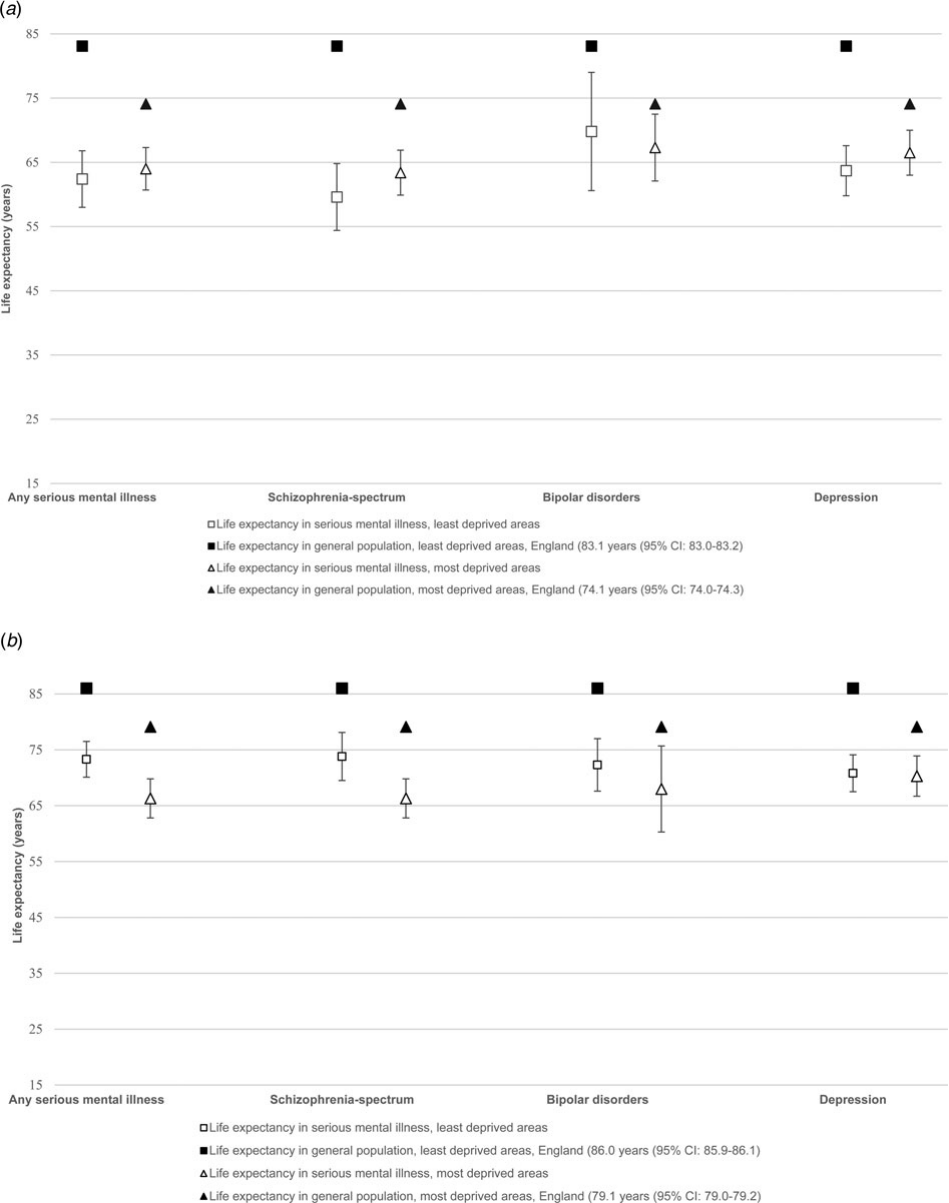
(*a*) Life expectancy in men with serious mental illness by area-level deprivation, (*b*) Life expectancy in women with serious mental illness by area-level deprivation.

We also assessed life expectancy at birth among people with SMIs living in the least and most deprived areas (Figs 1*a* and 1*b* also see online Supplementary Material: Table S1). The gap in life expectancy at birth was largest for men with schizophrenia-spectrum disorders residing in the least deprived areas, this amounted to a life expectancy at birth of 59.6 years (95% CI 54.3–64.8), a 23.4-year reduction compared with men residing in comparable areas of affluence (online Supplementary Table S1). Life expectancy at birth was lower in people with SMIs across all diagnostic groups, compared with the general population residing in areas of comparable deprivation. Of note, across all SMI diagnoses, people with SMIs had a lower life expectancy at birth compared with the general population residing in the most deprived areas (Figs 1*a* and 1*b*).

The supplementary tables (online Supplementary Tables S2a and S2b) highlight causes of death. As we have described previously, most causes of deaths (80% of deaths in schizophrenia-spectrum and bipolar disorders and 85% of deaths in major depression) were from preventable physical causes, while 11% of deaths in schizophrenia-spectrum and bipolar disorders and 7% of deaths in major depression were from unnatural causes, including suicide (Das-Munshi et al., 2017b; Das-Munshi et al., 2018; Das-Munshi et al., 2019).

## Discussion

### Main findings and interpretation

Our study indicates two key findings. First, in keeping with previous work (Chang et al., 2011; Hjorthøj et al., 2017), our findings indicate that men and women with SMIs, from a large urban sample from the UK, experience marked reductions in life expectancy at birth compared to the general population. In the most affluent areas, this amounted to a 20-year difference in men with any SMI. However, the excess risk was noted across all psychiatric diagnoses surveyed in men and women, and included schizophrenia-spectrum disorders, bipolar disorders and depression managed in secondary mental healthcare. Marked reductions in life expectancy in people with SMIs were evident across all ethnic groups, including the White British group with SMIs, indicating an adverse impact of having a SMI on mortality outcomes.

In stratified analyses, life expectancy at birth among people with SMIs remained lower than the general population resident in areas with equivalent levels of deprivation, indicating an effect of SMIs *over and above* deprivation effects on mortality outcomes. This effect of deprivation on life expectancy appeared to be stronger in women with SMIs compared to men. In the UK general population, large differences have been noted with individuals residing in the most deprived areas experiencing a shorter life expectancy than those residing in the most affluent areas; this follows a strong social class gradient and has been highlighted as a major area of public health concern (Bennett et al., 2018). Therefore, our second finding – that life expectancy at birth in SMI populations is substantially lower than the life expectancy observed for the general population in the most deprived areas – may suggest that people with SMIs are ‘*off the scale of the social hierarchy completely*’ (Marmot, 2018), akin to other groups known to experience excess mortality and other extreme health inequalities, due to being marginalised and socially excluded (Aldridge et al., 2018).

People with SMIs may experience additional multiple adversities, beyond those experienced by the most deprived communities in the UK. Findings from previous research have indicated that people with SMIs are less likely to have timely access to healthcare resources (e.g. specialist treatment) (Mitchell & Lawrence, 2018). It was not possible to further test interactions between ethnicity and area deprivation in this study, as this would have led to samples below the recommended size for assessments of life expectancy (Eayres & Williams, 2004). As in the UK, ethnically dense areas also tend to be more deprived, area-level interactions with mortality in mental illnesses could be complex. Findings from previous studies have indicated that in ethnic minority groups with SMI, residency in areas of higher own group density may be associated with a reduced risk of death from a range of causes, which could be due to the health-protective effects of social networks, social support, community participation and protection from social isolation and social exclusion (Das-Munshi et al., 2019). Interactions with area-level deprivation for ethnic minority groups could be explored in future work.

### Strengths and limitations

A limitation of our study related to the lack of information on ethnicity in death certificates, which is not recorded in the UK. As a result of this, other investigators had to use innovative methods – for example UK investigators previously used data linkages to derive these for ethnic minority groups in Scotland (Gruer et al., 2016), which could be an approach also used in this area in future. This limitation meant that we were only able to compare observed mortality in each of the ethnic minority groups in the sample with SMIs to life expectancy in the general population, without the benefit of being able to take into account life expectancy by ethnicity in the general population. One possibility is that the life expectancy of each of the ethnic minority groups was lower to start with. Although we should be cautious in comparing ethnic minority groups across the UK who may have differing histories of migration and settlement as well as experiences of health inequalities, in the study using linked data from Scotland, the life expectancy of Irish and South Asian groups was similar to the ‘population standard’ from England used in the present study (Gruer et al., 2016). This may suggest that the differences seen in our study reflect the over-riding harm of severe mental illness on life expectancy, although more work assessing these interacting factors will be needed, particularly because adverse mental and physical health outcomes in each of the ethnic minority groups in the present study have also been well described (Das-Munshi et al., 2017a; Oduola et al., 2019; Oduola et al.).

A further limitation is that the data from this study were from a mainly urbanised location which may limit generalisability to other samples, for example covering rural areas. However, the broader evidence base indicates that premature mortality is still significant in people with SMIs irrespective of how urbanised their location of residence is (Das-Munshi et al., 2019; Phillips, Yang, Li, & Li, 2004), and so we may anticipate similar trends if the study could be repeated in populations residing in less urban locations. As this study used data from electronic health records, a further limitation may have been due to the quality of the data input into the care record by clinicians. We used a well-validated and reliable method to ascertain clinical diagnoses. However, for other measures such as ethnicity, although this had high levels of completion, it is possible that this was not always self-ascribed. We plan to assess this and other measures against other sources (e.g. through linkages) in future work. We did not assess comorbid alcohol and substance use disorders and did not have information on physical health comorbidities. Both issues may play an important role in accounting for premature mortality in people with severe mental illnesses and should be considered in future work.

Finally, we did not have data on country of birth, therefore it was not possible to assess the impact of generational status in ethnic minority groups on life expectancy estimates. This is an important issue as health-related behaviours such as tobacco use, as well as exercise and dietary practices leading to weight gain, have been shown in previous work to converge to that of White British reference groups in first-generation migrants with a longer duration of residence, or show convergence across generations in ethnic minority groups (Alidu & Grunfeld, 2018; Hawkins, Lamb, Cole, & Law, 2008; Smith, Kelly, & Nazroo, 2011). Acculturation refers to the process whereby health-related behaviours, attitudes and beliefs change when people of one culture come into contact with another. It is possible that acculturation in certain British ethnic minority groups has led to the adoption of health-related behaviours usually more prevalent in White British groups; this issue could be explored in future work.

Our study utilised 8 years of cohort data from an ethnically diverse region in the UK, and therefore the statistical power to detect differences by ethnicity, sex and diagnoses and by area-level deprivation was enhanced, albeit with the caveat that the methodologies utilised in this report result in estimates which were less precise with lower sample sizes (Eayres & Williams, 2004). The estimates and 95% confidence intervals for the smaller samples in this report should, as a result, be viewed with caution (Eayres & Williams, 2004) and may reflect random variations. The catchment area of the study represents a large and well-defined region within south London and as such, may have good generalisability to other metropolitan and urban samples elsewhere.

In the UK, healthcare is free at the point of contact and delivery and within the catchment area at the time of the study, the hospital Trust provided near-complete coverage of secondary mental healthcare services. Therefore, we can be reasonably certain that our samples for schizophrenia-spectrum and bipolar disorders are fairly representative of all individuals with the condition living in the catchment area since it is highly likely that such individuals would have made contact with secondary mental healthcare services at some point over the course of their illness. However, as most cases of depression in the UK are managed in primary care, our sample of people with depression represent the more ‘severe’ end of the spectrum, who may present challenges to standard management in primary care, for example through treatment resistance or comorbidities (Das-Munshi et al., 2018). Therefore, the estimates for reduced life expectancy in the depression sample in this study should be viewed as those representing people with more complex/severe conditions in contact with secondary mental healthcare services and may not reflect estimated life expectancy for people with depression solely managed in primary care, or never treated.

The linkage to death certificates would have meant that deaths occurring anywhere in England and Wales (even if outside of the immediate catchment area of the mental health Trust) would have been captured. In previous analyses we have also used the linked data on mortality and known emigrations to model the possibility that migrant groups may be more likely to emigrate and potentially die outside of the UK, leading to a numerator-denominator mismatch and possibly artefactually lowering standardised mortality rates (Razum, 2006). Our previous work which has taken this data on emigrations into account has indicated estimates for analyses relating to deaths to be robust to this possibility of out-migration (Das-Munshi et al., 2017b, 2018).

### Implications

There are longstanding concerns around the corrosive effects of health inequalities across the social gradient or hierarchy in the general population (Institute of Health Equity, 2010; Marmot, Allen, Boyce, Goldblatt, & Morrison, 2020). Yet these findings highlight that life expectancy at birth among people with SMIs, irrespective of ethnicity, is lower and more adversely impacted upon than the most deprived communities in the UK. Put simply, people with SMIs experience excess mortality greater than those who are already on the lowest rung of the ‘social ladder’. The larger excess risk of death (around 20 years) in men with any SMI, schizophrenia-spectrum disorders and major depression, in the most affluent areas, compared to men in the general population, further brings into focus the extent of this stark inequality. Future work may need to broaden from a focus on traditional risk factors (smoking, weight and other cardiovascular risks) to approaches which enhance social inclusion and address multiple intersecting disadvantages and structural inequalities (Marmot, 2018).

The findings, therefore, quantify the extent to which public health, as well as the health and social care systems, continue to fail to address this important measure of health inequality in people with SMIs. Addressing structural inequalities such as poverty, improving equitable access to healthcare (Bennett et al., 2018) and tackling causes with a focus on individuals, communities and health systems (Liu et al., 2017) and strengthening approaches towards evidence-based clinical practice for common preventable physical health conditions (World Health Organization, 2018) are required, alongside a consideration of those aspects which promote the social inclusion of people with SMIs.

## Data Availability

Data are owned by a third party SLaM BRC CRIS tool which provides access to anonymised data derived from SLaM electronic medical records. These data can only be accessed by permitted individuals from within a secure firewall (i.e. remote access is not possible and the data cannot be sent elsewhere), in the same manner as the authors.

## Supplementary material

For supplementary material accompanying this paper visit https://doi.org/10.1017/S0033291720001087. click here to view supplementary material
