# Supplementary material for: How do ethnicity and deprivation impact on life expectancy at birth in people with serious mental illness? Observational study in the UK
Source: Psychol Med. 2020 May 6;51(15):2581–9. doi: 10.1017/S0033291720001087 (PMC8579155; doi:10.1017/S0033291720001087)
Supplement: Supplementary file 1 [file S0033291720001087sup001.docx]

**SUPPLEMENTARY MATERIAL**

**Supplementary Table 1: Deaths in men and women with serious mental disorder diagnoses, residing in the least/ most deprived areas**

|  |  | **Men** |  |  |  | **Women** |  |  |
| --- | --- | --- | --- | --- | --- | --- | --- | --- |
| **Diagnosis** |  | **Deaths/ total** | **Life expectancy (95% CI)** | **Difference from men in general population residing in comparable areas*** |  | **Deaths/ total** | **Life expectancy (95% CI)** | **Difference from women in general population residing in comparable areas**** |
| **Least deprived decile** |  |  |  |  |  |  |  |  |
| Any serious mental illness |  | 82/ 864 | 62.4 (58.1, 66.8) | -20.7 |  | 87/ 910 | 73.3 (70.1, 76.6) | -12.7 |
| Non-affective (schizophrenia-spectrum) |  | 65/ 579 | 59.6 (54.3, 64.8) | -23.5 |  | 54/ 475 | 73.8 (69.6, 78.1) | -12.2 |
| Affective (Bipolar disorders) |  | 17/ 285 | 69.8 (60.4, 79.1) | -13.3 |  | 33/ 435 | 72.3 (67.7, 77.0) | -13.7 |
| Depression |  | 98/ 783 | 63.7 (59.9, 67.6) | -19.4 |  | 114/ 1251 | 70.8 (67.6, 74.1) | -15.2 |
| **Most deprived decile** |  |  |  |  |  |  |  |  |
| Any serious mental illness |  | 96/ 1020 | 64.0 (60.8, 67.3) | -10.1 |  | 90/ 780 | 66.3 (62,7, 69.8) | -12.8 |
| Non-affective (schizophrenia-spectrum) |  | 83/873 | 63.4 (59.8, 66.9) | -10.7 |  | 73/ 564 | 65.6 (61.7, 69.5) | -14.1 |
| Affective (Bipolar disorders) |  | 13/ 147 | 67.3 (62.1, 72.5) | -6.8 |  | 17/ 216 | 68.0 (60.2, 75.7) | -11.1 |
| Depression |  | 84/ 722 | 66.5 (63.0, 70.0) | -7.6 |  | 131/ 1278 | 70.3 (66.7, 73.9) | -8.8 |

*Key: * Life expectancy for men in the general population was 74.1 years (95% CI: 74.0-74.1)in the most deprived IMD decile and 83.1 (95% CI 83.0-83.2) years in the least deprived IMD deciles in England , 2011 to 2013; **Life expectancy for women in the general population was 79.1 years (95% CI: 79.0-79.2) in the most deprived IMD decile and 86.0 years (95% CI: 85.9-86.1) in the least deprived deciles in England , 2011 to 2013*

**Supplementary tables 2:** Causes of death across samples

**Table 2a:** **Deaths by cause across sample**

(F2*, F30, F31 (schizophrenia spectrum and bipolar diagnoses)

|  | **N** | **%** |
| --- | --- | --- |
| *Total sample* | *18,641* | *100* |
| Alive* | 16855 | 90 |
| Died- natural causes | 1430 | 8 |
| Died- unnatural causes | 197 | 1 |
| Died- unknown causes | 159 | 1 |

**includes 1094 emigrations*

**Table 2b:** **Deaths by cause across sample**

(F32, F33 (major depression diagnoses)

|  | **N** | **%** |
| --- | --- | --- |
| *Total sample* | *20,203* | *100* |
| Alive* | 17,842 | 88 |
| Died- natural causes | 1996 | 10 |
| Died- unnatural causes | 170 | 1 |
| Died- unknown causes | 195 | 1 |

**includes 609 emigrations*
